# Laryngeal Preservation Strategies in Locally Advanced Laryngeal and Hypopharyngeal Cancers

**DOI:** 10.3389/fonc.2019.00419

**Published:** 2019-05-31

**Authors:** Athanassios Argiris, Jean Louis Lefebvre

**Affiliations:** ^1^Department of Medical Oncology, Thomas Jefferson University, Philadelphia, PA, United States; ^2^Centre Oscar Lambret, Lille, France

**Keywords:** laryngeal preservation, surgery, radiotherapy, chemotherapy, biotherapy

## Abstract

For long, the treatment of locoregionally advanced laryngeal and hypopharyngeal squamous cell cancers (SCC) consisted of either total laryngectomy (TL) or definitive radiotherapy (RT). The development of induction cisplatin plus 5-fluorouracil (PF) and the correlation between chemosensitivity and radiosensitivity in previously untreated patients opened a new era of treatment aiming at laryngeal preservation (LP). The fundamental concept was to employ induction PF in order to select patients for subsequent treatment with either TL or RT according to tumor response to PF. The first two trials (VALGSG for laryngeal SCC and EORTC 24891 for hypopharyngeal SCC) concluded that such an approach could preserve nearly 60% of larynx without deleterious impact on survival. The EORTC 24954 trial compared 4 cycles of induction PF followed by RT in good responders vs. alternating PF-RT in laryngeal and hypopharyngeal SCC. There was no significant difference in 5-year overall survival with a functional larynx between the two arms (31 vs. 35%). The GORTEC 2000-01 trial compared induction PF to induction PF plus docetaxel (TPF) both followed by RT in good responders in larynx and hypopharynx SCC. The 5-year LP was significantly higher in the TPF arm (60 vs. 39%) but without a difference in survival. The RTOG 91-11 trial compared induction PF followed by RT in good responders vs. concurrent chemoradiotherapy (chemo-RT) vs. RT alone in laryngeal SCC. There was no significant difference in 5-year laryngectomy-free survival between the patients treated with induction chemotherapy (44%) vs. those treated with chemo-RT (47%), both being superior to RT alone (34%). At 5 years, LP was superior with chemo-RT: 84 vs. 71% with induction PF. Two phase II trials explored the role of cetuximab (E) in LP in laryngeal and hypopharyngeal SCC. The TREMPLIN trial compared RT+E or chemo-RT (RT + P) after TPF. The DeLOS-II trial compared TPE followed by RT+E vs. TP followed by RT. However, these trials failed to indicate an advantage for the incorporation of E in the treatment paradigm. To date, two approaches for LP have been validated: induction TPF followed by RT for laryngeal and hypopharyngeal SCC and concurrent chemo-RT for laryngeal SCC. An ongoing trial (SALTORL) is comparing these two approaches, induction TPF and chemo-RT, in laryngeal/ hypopharyngeal SCC.

## Introduction

Since the beginning of the twentieth^.^ century two major options were available for the treatment of locally advanced laryngeal and hypopharyngeal squamous cell carcinomas (SCC): definitive radiation therapy (RT) with salvage surgery reserved in case of local failure or total laryngectomy with postoperative RT. The indications for each approach varied according to institutional policies. Since no randomized trials with these two approaches were available at that time and results were derived from retrospective analyses comparisons of outcomes and the merits of each treatment strategy were highly debatable.

For long, clinical investigations aimed at extending the indications of partial laryngectomy or exploring different protocols of RT using altered fractionation schedules or concurrent radiosensitizers. These efforts did not notably alter the main treatment approaches (i.e., surgical vs. non-surgical) in laryngeal and hypopharyngeal SCC. At that time chemotherapy was mainly used for the palliative treatment of head and neck SCC.

An important milestone was the publication in 1983 by the Wayne State University of its experience with induction chemotherapy using cisplatin and 5-fluorouracil (PF) in previously untreated patients with head and neck cancers. In a series of 35 patients treated with three cycles of induction chemotherapy with PF, 94% demonstrated a tumor reduction of at least 50 and 63% had a complete clinical disappearance of the disease (1). In another report on 60 patients treated by induction cisplatin-based chemotherapy it appeared that the 42 patients who had demonstrated a tumor response over 50%, 97% of them were controlled by a subsequent RT opposite to 6% of the 18 patients with a tumor reduction below 50% who were controlled by a subsequent RT (2). For the first time, induction chemotherapy was shown to have a potential role in curative intent treatment and could assist in selecting good candidates for subsequent definitive RT. These data re-opened the discussion on the treatment of advanced laryngeal and hypopharyngeal cancers. Two approaches were under discussion: (a) induction PF followed by RT in good responders (tumor regression of at least 50%) or by surgery in other patients and (b) upfront surgery and postoperative RT. Later on, the results of a large meta-analysis showed that concurrent chemoradiotherapy (CRT), in particular with cisplatin-based regimens, achieved better results in terms of survival than induction PF followed by RT (3). Finally, the introduction of induction PF plus docetaxel (TPF protocol) and the use of cetuximab enriched the potential clinical research questions. Several clinical protocols explored induction chemotherapy, concurrent CRT, and the combination of these two approaches.

## The First Trials With Induction Chemotherapy

The main objective of these phase III trials was to compare upfront total laryngectomy with postoperative RT with an experimental approach with induction PF followed in responders by RT (with salvage surgery if required for failures after RT) or by a total laryngectomy with postoperative RT in non-responders.

Each cycle of chemotherapy consisted of cisplatin 100 mg/m^2^ on day 1 followed by 5-fluorouracil 1,000 mg/m^2^ /day for 5 days and was delivered every 3 weeks. Definitive RT was administered to a total dose of 70 Gy and postoperative RT to a total dose of 60 Gy. “Responders” to chemotherapy were defined as patients with a tumor regression of at least 50%.

The primary end-point was under discussion as these first trials were designed. Published data from surgical series provided good results in terms of survival and locoregional control but on selected patients (operable patients with resectable disease). The reported survival rates after definitive RT were lower but included patients with worse prognosis (e.g., unresectable or inoperable). To validate the concept of laryngeal preservation the prerequisite was to assure that there was no deleterious impact on disease control and survival. Therefore, the two first trials had survival as their primary end-point. However improving overall survival has not been a primary objective given the impact of salvage surgery on overall survival.

### The Veterans Administration Larynx Cancer Study Group (VALCSG) Trial

In the United States, the department of VALCSG conducted this randomized trial in 332 laryngeal cancer patients (166 in the surgical control arm and 166 in the experimental arm) (4). The experimental treatment consisted of two cycles of PF followed in responders by a third cycle and RT or surgery and postoperative RT in non-responders). Overall survival was the primary endpoint. At a median follow-up of 33 months, the 2-year survival was 68% in both treatment arms (95% Confidence Interval [CI]: 60–75% in the surgery arm vs. 60–76% in the chemotherapy arm, *P* = 0.9846) and the larynx was preserved in 64% of the patients in the experimental arm. In the chemotherapy arm, salvage laryngectomies were indicated significantly more often in patients with T4 diseases vs. those with T3 disease (*P* = 0.001). Of note distant metastases were observed less frequently in the chemotherapy arm (4).

### The European Organization for Research and Treatment of Cancer (EORTC) 24891 Trial

In Europe, the EORTC Head and Neck Cooperative Group conducted a similar trial in patients with advanced hypopharyngeal and lateral epilarynx tumors requiring a total laryngectomy (5). In this EORTC 24891 trial, 194 previously untreated patients were enrolled.

Chemotherapy consisted of 100 mg/m^2^ given intravenously over a 1-h period followed by fluorouracil 1,000/m^2^ /day given as a 120-h infusion over 5 days (total dose 5,000 mg/m^2^). A partial response (PR) after two or three cycles of chemotherapy was required to receive RT. The primary endpoint was overall survival in terms of non-inferiority in the experimental arm with a hazard ratio (HR) ≤ 1.43. In the first evaluation the median duration of survival was 25 months in the immediate-surgery arm and 44 months in the induction-chemotherapy arm and, since the observed hazard ratio was 0.86 (log-rank test, *P* = 0.006), which was significantly < 1.43, the two treatments were judged to be equivalent. The 3- and 5-year estimates of retaining a functional larynx in patients treated in the induction-chemotherapy arm were 42% (95% CI: 31–53%) and 35% (95% CI: 22–48%), respectively (5).

These results were confirmed by long-term evaluation. At a median follow-up of 10.5 years, the 5-year and 10-year overall survival rates were, respectively, 32.6% (95% CI: 23.0–42.1%) and 13.8% (95% CI: 6.1–21.6%) in the surgery arm vs. 38.0% (95% CI: 28.4–47.6%) and 13.1% (95% CI: 5.6–20.6%) in the chemotherapy arm. In 37 patients still alive at 5 years in the chemotherapy arm, 22 (59.5%) had retained a normal larynx (6). It is noteworthy that distant metastases were less frequent in the chemotherapy arm as in the American trial.

### Conclusions After These Trials

These two trials showed that the concept could be validated, both for laryngeal and hypopharyngeal cancers, as the larynx could be preserved in about two-thirds of the patients without compromising survival or disease control. This clinical research paradigm, therefore, could continue with the primary end-point being laryngeal preservation. However, the definition of “laryngeal preservation” had to be clearly defined.

Laryngeal preservation may be defined by only one parameter: larynx in place (i.e., no laryngectomy). A more comprehensive one is to consider both the organ and its function: no laryngectomy, no long-term tracheotomy, and no long-term feeding tube, which implies also that local control is obtained. As survival is an important issue, it may also be integrated in the definition of laryngectomy-free survival or survival with a functional larynx in place.

In 2009, a group of experts fine-tuned the definition of laryngeal preservation taking into account all parameters participating to the real benefit for the patients. They elaborated the “laryngoesophageal dysfunction-free survival” that combined as events: death, local failure, salvage laryngectomy, and tracheotomy or feeding tube at 2 years or later (7, 8).

### The EORTC 24954 Trial

The EORTC Head and Neck and Radiotherapy Oncology Cooperative Groups designed a randomized trial in order to compare two different schedules for delivering more cycles of chemotherapy: a sequential schedule like the one used in the previous EORTC 24891 trial vs. an alternating one as described by Merlano (9). The sequential arm consisted of two cycles of PF with the same doses and administration as in the 24891 trial. After 2 cycles responders received two additional cycles of PF and were then treated with RT at a dose of 70 Gy. The non-responders were treated by total laryngectomy and postoperative RT. In the alternating arm, patients received on weeks 1, 4, 7, and 10 a cycle of chemotherapy consisting of cisplatin at a dose of 20 mg/m^2^ per day on days 1–5 (for a total dose of 100 mg/m^2^) and 5-fluorouracil by bolus infusion at a dose of 200 mg/m^2^ per day on days 1–5 (for a total of 1,000 mg/m^2^). During the three 2-week intervals patients were treated by RT at a dose of 20 Gy per course for a total of 60 Gy. As a result, the total doses of 5-fluorouracil and of RT were lower in the alternating arm. A total of 450 patients were enrolled in this trial (224 to the sequential arm and 226 to the alternating arm).

For the first evaluation the median follow-up was 6.5 years. Survival with a functional larynx was similar in the sequential and alternating arms (hazard ratio of death and/or event = 0.85, (95% CI: 0.68–1.06), as were median overall survival (4.4 and 5.1 years, respectively). Grade 3 or 4 mucositis occurred in 64 (32%) of the 200 patients in the sequential arm who received radiotherapy and in 47 (21%) of the 220 patients in the alternating arm. Late severe oedema and/or fibrosis was observed in 32 (16%) patients in the sequential arm and in 25 (11%) in the alternating arm (10).

For the long-term evaluation, the median follow-up was 10.2 years. Ten-year survival with a functional larynx (primary end-point) and overall survival were similar in the sequential and alternating arms (18.7 and 33.6% vs. 18.3 and 31.6%, respectively). Late toxicity was also similar even if there was a trend for higher laryngeal preservation and better laryngeal function in the alternating arm (11). The lower doses of chemotherapy and RT in the alternating arm may explain the better tolerance to treatment. However, due to the organizational difficulties when delivering such an alternating schedule in daily practice, it is rarely used.

### The Groupe Oncologie Radiotherapie Tete Et Cou (GORTEC) 2000-01 Trial With Cisplatin, 5-FU, Docetaxel

Two large randomized trials (12, 13) had shown that adding docetaxel to cisplatin fluorouracil (the so-called TPF regimen) before RT (or CRT) resulted in a significantly higher survival compared to that observed with the doublet regimen (PF).

In France, in order to assess whether induction TPF could provide better results than induction PF in the frame of laryngeal preservation, the GORTEC conducted a two-arm randomized trial in 220 patients with a locally advanced laryngeal or hypopharyngeal cancer eligible for a total laryngectomy. Patients were randomized between an experimental arm starting with TPF (docetaxel at 75 mg/m^2^ on day 1, cisplatin at 75 mg/m^2^ on day 1, and 5-fluorouracil at a dose of 750 mg/m^2^ by 120-h continuous infusion over 5 days) compared with the classical PF one (cisplatin 100 mg/m^2^ on day 1 and 5-fluorouracil given at a dose of 1,000 mg/m^2^ by 120-h continuous infusion over 5 days). Three cycles at a 3-week interval were planned in the two arms and responders were treated by RT while non-responders had total laryngectomy and postoperative RT. Laryngeal preservation (larynx in place without tumor, tracheostomy or feeding tube) was the primary end-point. Overall survival and progression-free survival were secondary endpoints. two hundred twenty patients were enrolled, of whom 213 were eligible (110 in the TPF arm and 103 in the PF arm).

The first evaluation revealed that in the TPF arm 69 patients (62.7%) could receive the complete treatment without delay or dose reduction vs. 33 patients (32%) in the PF arm. The response rates were 80% with TPF arm and 59.2% with PF (*P* = 0.002). As a result, laryngeal preservation was offered to 78.8% of patients in the TPF arm vs. 55.3% in the PF arm. With a median follow-up of 36 months, the 3-year actuarial laryngeal preservation rate was 70.3% in the TPF arm vs. 57.5% in the PF arm (*P* = 0.002) (Table 1). However, there were no significant differences in terms of survival (14).

**Table 1:** Key phase III trials of laryngeal preservation strategies.

**Study**	**Patients enrolled**	**Investigational regimen(s)**	**Control regimen**	**Primary endpoint(s)**	**Results**	**Comments**
RTOG 91-11	547 (larynx 100%)	a) CRT c) RT	b) PF followed by RT	Laryngeal preservation Laryngectomy-free survival	Laryngeal preservation rates at 5 years (a vs. b vs. c): 84% vs. 71% vs. 66% Laryngectomy-free survival at 5 years (a vs. b vs. c): 47% vs. 44% vs. 34% Overall survival at 5 years (a vs. b vs. c): 55% vs. 58% vs. 54%	CRT and PF followed by RT are superior to RT alone
GORTEC 2000-01	213 (larynx, 46%; hypopharynx, 54%)	TPF followed by RT^*^	PF followed by RT^*^	Laryngeal preservation	Laryngeal preservation rates (actuarial) at 3 years: 70.3 vs. 57.5% No difference in overall survival (60% at 3 years in both arms)	TPF is more effective than PF

* *16% of patients in the PF arm and 20% of patients in the TPF arm received concurrent chemotherapy during RT. CRT, chemoradiotherapy; RT, radiotherapy; TPF, cisplatin, docetaxel, 5-fluorouracil; PF, cisplatin, 5-fluorouracil*.

The long-term evaluation confirmed the initial results. The 5-year and 10-year laryngeal preservation rates were 74.0% (95% CI: 64–82%) vs. 58.1% (95% CI: 47–68%) and 70.3% (95% CI: 58–80%) vs. 46.5% (95% CI: 31–63%, *P* = 0.01) with TPF and PF, respectively. There was no significant difference in 5-year and 10-year overall survival, or disease-free survival. Of note there were fewer grade 3–4 late toxicities in the TPF arm (9.3%) than in the PF arm (17.1%, *P* = 0.038) (15).

Of note, in this trial it was left to institutional policies to deliver either radiotherapy alone or concurrent chemoradiotherapy in responders. Seventeen patients in the TPF arm and 9 patients in the PF arm received concurrent chemo-radiation. The impact of this on the overall study results is unknown.

## The Radiation Therapy Oncology Group (RTOG) 91-11 Trial with Concurrent Chemoradiotherapy

In the Unites States, the RTOG and the Head and Neck Intergroup conducted a three-arm randomized trial comparing the standard alternative to total laryngectomy validated by previous trials (induction PF chemotherapy followed by radiotherapy) vs. radiotherapy with concurrent cisplatin vs. radiotherapy alone in 547 previously untreated patients with locally advanced larynx cancer (16). Laryngectomy-free survival was the primary endpoint while laryngeal preservation (larynx in place) and survival were secondary endpoints. This study excluded patients with large-volume stage T4 disease defined as tumor penetrating through the cartilage or extending more than 1 cm into the base of tongue. In total only 10% of patients enrolled in 91–11 trial had stage T4 tumors.

In the first report no difference was found in acute toxicity during the radiotherapy between the induction chemotherapy and the radiotherapy alone arm. The 2-year and the 5-year estimates for laryngectomy-free survival were, respectively, 59 and 43% in the induction arm, 66 and 45% in the concurrent arm, and 53 and 38% in the radiotherapy alone arm. The difference was not significant between the induction and the concurrent arms. The 2-year and 5-year overall survival did not differ significantly according to the treatment arm. The rate of laryngeal preservation at a median follow-up of 3.8 years was significantly higher in the concurrent arm (84%) when compared with the induction arm (72%, *P* = 0.005) or with the radiotherapy alone arm (67%, *P* < 0.001) (16).

The long-term analysis with a median follow-up of 10.8 years in surviving patients confirmed that there was no significant difference in late toxicity between the three arms. The two chemotherapy arms significantly improved laryngectomy-free survival compared with radiotherapy alone without significant difference between these two arms. Overall survival did not differ significantly between the treatment arms, although there was a trend for a higher survival in the induction arm. However the rate of deaths not related to the study cancer was significantly higher in the concurrent arm compared with the induction one (69.8 vs. 52.8%, respectively, at 10 years, *P* = 0.03). With regards to laryngeal preservation, the difference favoring the concurrent arm with regards to the laryngeal preservation persisted at 10 years 67.5% (95% CI: 60.4–74.6%) in the induction arm, 81.7% (95% CI: 75.9–87.6%) in the concurrent arm, and 63.8% (95% CI: 56.5–71.1%) in the radiotherapy alone arm (17) (Table 1). Again, there were fewer distant metastases in the two arms with chemotherapy when compared with radiotherapy alone.

Long-term results of 91–11 confirm that CRT is a standard treatment option but also raise concerns about late effects from CRT leading to increased number of non-cancer related deaths.

## Trials Integrating Cetuximab and Combining Induction Chemotherapy Followed by Concurrent Chemoradiotherapy

A randomized trial had shown that adding cetuximab to RT significantly provided higher survival and loco-regional control over RT alone (18). Therefore, further study of cetuximab in combined modality regimens was worth exploring.

### The GORTEC “TREMPLIN Trial”

An experimental approach with induction chemotherapy followed by concurrent CRT was tested in the laryngeal preservation setting. Anticipating an overall toxicity that could compromize the larynx function, and taking into account the results of the radiotherapy plus cetuximab trial (18), the GORTEC conducted a randomized phase II study to assess what could be the best post-induction protocol in 153 patients with laryngeal or hypopharyngeal cancer amenable to a total laryngectomy (19).

Patients received 3 cycles of TPF and responders were randomized between RT plus cisplatin (100 mg/m^2^ on day 1, 22, and 43 of RT) and RT plus cetuximab (a loading dose of 400 and 250 mg/m^2^ per week during RT. The primary endpoint was laryngeal preservation (no residual disease justifying immediate salvage laryngectomy) 3 months after the end of treatment. The secondary endpoints were larynx function preservation and overall survival 18 months after the end of treatment.

Of the 153 enrolled patients, 116 were randomized (60 in the cisplatin arm, and 56 in the cetuximab arm). Substantial acute toxicity was observed in both arms, in particular in-field skin toxicity in the cetuximab arm and renal, hematological, and performance status alteration in the cisplatin arm. Limiting acute toxicity led to protocol modification in more patients in the cisplatin arm than in the cetuximab arm (71 and 43 vs. 71%, respectively). Except for grade 1 renal toxicity, late toxicity did not differ significantly between both arms. At last examination, there were fewer local recurrences in the cisplatin arm (8 patients) compared with 12 patients in the cetuximab arm, but successful salvage surgery could be performed only in the cetuximab arm.

There was no significant difference in laryngeal preservation at 3 months: 95% (95% CI: 86–98%) in the cisplatin arm vs. 93% (95% CI: 83–97%) in the cetuximab arm. There was no obvious difference in secondary endpoints at 18 months as well. The larynx function preservation was 87% (95% CI: 76–93%) in the cisplatin arm vs. 82% (95% CI: 70–90%) in the cetuximab arm. The overall survival was 92% in the cisplatin arm (95% CI: 82–96%) and 89% (95% CI: 79–95%). At a median follow-up of 36 months overall survival was 75% (95% CI: 62–85%) and 73% (95% CI: 60–84%) in the cisplatin arm and cetuximab arm, respectively. These data must be considered with caution as they related to the population selected after induction chemotherapy (i.e., 75% of the overall population).

As the composite end-point of laryngoesophageal dysfunction-free survival had been described after the trial was initiated and had been published at the time of the trial evaluation, this end-point was tested. Two years after the end of treatment there was no significant difference in that end-point: 79% (95% CI: 67–89%) with cisplatin vs. 72% (95% CI: 65–89%) with cetuximab (19).

The conclusion was that there was no signal that one arm was superior over the other one, and none appeared to be superior to induction TPF followed by RT alone as found in the above-mentioned GORTEC 2000-01 trial.

After induction TPF it is difficult to administer high-dose cisplatin due to cumulative toxicities. RT plus carboplatin or cetuximab have been explored but we do not have any data coming from trials specifically designed for laryngeal preservation. However, whether the addition of a systemic agent to RT after TPF induction is superior to RT alone is unproven.

### The German “DeLOS-II Trial”

The German Larynx Organ preservation Study group (DeLOS) conducted another randomized phase II study assessing the place of cetuximab in laryngeal preservation for patients with larynx or hypopharynx cancer (20). The initial trial design was to compare induction TPF followed by RT with TPF plus cetuximab (E) followed by RT plus cetuximab. Due to 4 treatment-related deaths among the first 64 patients, the protocol was amended and fluorouracil was omitted from induction chemotherapy in both arms. There were no further treatment-related deaths thereafter. The evaluation was made after one cycle and responders continued the protocol while non-responders went to laryngectomy. The primary objective was a 2-year functional laryngectomy-free survival (fLFS) above 35%.

Of the 180 patients randomized in the trial, 173 fulfilled the intent to treat criteria. At final examination, the objective response rates in the arm without cetuximab were 79.1% in patients who had received PF, and 94.7% in patients who had received TP. In the arm with cetuximab they were 80% in patients who had received TPFE, and 94.9% in patients with TPE, 94.9% (i.e., similar to TPF). The primary objective was similarly met in both arms: 44.7% in the arm without cetuximab and 46.6% in the cetuximab arm (OR:0.9268, 95% CI:0.5094–1.6863). There was no difference in 2-year overall survival: 68.2% in the arm without cetuximab, and 69.3% in the cetuximab arm (OR:0.9508, 95% CI:0.4997–1.8091).

The conclusions were that despite being accompanied by an elevated frequency in adverse events, the induction chemotherapy with TPF/TP plus cetuximab was feasible but showed no superiority to induction chemotherapy with TPF/PF alone regarding LFS and OS at 24 months (20).

## Conclusions

To date, only two strategies for laryngeal preservation in previously untreated patients with locally advanced laryngeal and hypopharyngeal cancers have been validated: induction TPF followed by RT alone (GORTEC 2000-01) and RT with concurrent cisplatin (RTOG 91-11). Whereas, both approaches have been assessed in laryngeal cancers, only induction chemotherapy-based protocols have been evaluated in hypopharyngeal cancers. The RTOG 91–11 trial did not contain an arm with TPF induction as this trial was initiated before the TPF induction regimen was proved to be superior to PF in the GORTEC 2000-01 trial. As a result, there is a need to compare the RTOG concurrent arm and the TPF arm of the GORTEC trial. The ongoing French phase III trial (GORTEC 2014-03-SALTORL, clinicaltrials.gov NCT03340896) is comparing induction TPF followed by RT in responders vs. concurrent cisplatin-based chemoradiotherapy with the composite end-point of laryngoesophageal dysfunction-free survival as primary end-point. Eligible are patients with stage T2-3, N0-2 laryngeal, or hypopharyngeal SCC requiring total laryngectomy. Patients with pretreatment poor laryngo-esophageal function (in particular those requiring a pre-treatment tracheostomy) should be treated by upfront TL.

The decision of enrolling a patient in a laryngeal preservation protocol must be taken by a multidisciplinary tumor board. We acknowledge that there is significant variability between centers worldwide regarding the applicability of clinical trial results on laryngeal preservation approaches. In general, patients eligible for a laryngeal preservation strategy are patients with advanced larynx and hypopharynx cancers who are not eligible for partial surgery. Of importance, bulky T4 tumors extending to the post-cricoid area are not eligible for laryngeal preservation. Also, patients who are not candidates to receive cisplatin should not be generally be considered for a laryngeal preservation approach given the low success rates with RT alone. Treatment with RT plus cetuximab is not a validated approach for laryngeal preservation and may result in inferior outcomes compared to RT plus cisplatin.

To transition the outcomes of these trials into clinical practice it is important to strictly follow the study protocols with respect to initial work-up and eligibility criteria, chemotherapy protocols, prophylaxis/management of treatment-induced toxicity, response to treatment evaluation, as well as schedule and tools for post-treatment follow-up. Such approaches require experienced multidisciplinary teams.

## Author Contributions

All authors listed have made a substantial, direct and intellectual contribution to the work, and approved it for publication.

### Conflict of Interest Statement

JL has been member of the advisory boards of Sanofi- Aventis (docetaxel) and Merck Serono (cetuximab). JL has been lecturer for Sanofi-Aventis and Merck Serono. AA has been a member of advisory board and lecturer for Merck Serono (cetuximab).
